# Tissue-specific metabolomic profiling reveals cultivar-dependent drought tolerance mechanisms in rice (*Oryza sativa* L.)

**DOI:** 10.1186/s12870-025-08062-9

**Published:** 2026-01-21

**Authors:** Sobhi F Lamlom, Nagy S. Radwan, Abdul-Hamid Emwas, Mariusz Jaremko, Nader R. Abdelsalam

**Affiliations:** 1 Department of Plant Production, Faculty of Agriculture Saba Basha, Alexandria University, Alexandria, Egypt; 2 Agricultural Botany Department, Faculty of Agriculture, Alexandria University, 21531 Saba Basha, Egypt; 3 Core Lab of NMR, King Abdullah University of Science and Technology (KAUST), Thuwal, 23955-6900 Makkah Saudi Arabia; 4 Smart-Health Initiative (SHI) and Red Sea Research Center (RSRC), Division of Biological and Environmental Sciences and Engineering (BESE), King Abdullah University of Science and Technology (KAUST), Thuwal, 23955-6900 Makkah Saudi Arabia

**Keywords:** Rice, Drought stress, Metabolites, GC-MS, Cultivar tolerance, Osmotic adjustment

## Abstract

Drought stress threatens rice production globally, causing yield reductions of 50–70%. This study employed gas chromatography–mass spectrometry (GC-MS)-based metabolomics to investigate drought responses in four Egyptian rice cultivars (Giza 177, Giza 178, Sakha 104, Sakha 108) exposed to 15% polyethylene glycol stress for 14 days. Sakha 104 demonstrated the highest drought tolerance index (82.7%). Metabolomic analysis identified 114 polar metabolites in leaves and 97 in roots, representing amino acids, organic acids, sugars, and related compounds involved in osmotic adjustment. Principal component analysis (PCA) showed greater metabolic adaptability in roots (70.4% variance) than leaves (53.9%). Variable importance in projection analysis identified 40 metabolites with VIP > 1.0, among which 13 priority biomarkers (VIP > 1.6) showed the strongest discriminatory capacity, including amino acids, organic acids, sugars, and secondary metabolites. Hierarchical clustering revealed leaf metabolomes grouped by treatment, while root metabolomes clustered by cultivar, indicating tissue-specific adaptation strategies. Cultivar-specific signatures included leucine and L-serine in Giza 177, increased adenosine-5’-monophosphate in Sakha 104, higher oxalic acid and spermine in Sakha 108, and shikimic acid in Giza 178 roots. Venn diagram analysis identified only four metabolites universally expressed in leaves and three in roots. Strong correlations between metabolomic profiles and physiological traits confirmed the relevance of the biomarkers. Root tissues exhibited roughly 20 times more metabolic diversity than leaves, supporting their role as primary stress sensors. These findings identify practical metabolic biomarkers for breeding programs and shed light on the biochemical mechanisms underlying drought tolerance in Egyptian rice germplasm.

**Supplementary Information:**

The online version contains supplementary material available at 10.1186/s12870-025-08062-9.

## Introduction

Rice (*Oryza sativa* L.) constitutes the cornerstone of global food security, providing sustenance for more than 3.5 billion people and serving as the principal caloric source for over 50% of the world’s population [1, 2]. In Egypt and North Africa, where rice cultivation supports millions of livelihoods and contributes significantly to food security, drought poses an escalating economic threat. Egypt, Africa’s largest rice producer, has experienced yield losses exceeding 40% during drought years, resulting in annual economic losses of hundreds of millions of dollars [3, 4]. Climate projections indicate that water scarcity will intensify across the Nile Delta and North African rice-growing regions, with drought frequency expected to increase by 20–30% by 2050 [5, 6]. This creates an urgent imperative to understand the biochemical mechanisms underlying drought tolerance in locally adapted cultivars and to develop resilient varieties capable of maintaining productivity under increasingly water-limited conditions.

Drought stress is the most significant abiotic factor limiting rice production worldwide, with yield reductions of 50–70% often reported under severe water shortages conditions [7, 8]. Unlike other cereals, rice evolved mainly in aquatic and semi-aquatic habitats, leading to physiological traits designed for constant flooding or waterlogged soils [9, 10]. This evolutionary heritage makes rice highly vulnerable to water scarcity, requiring about 1,432 L of water to produce 1 kg of grain, which significantly surpasses the water needs of wheat, maize, or sorghum [11]. As a result, developing drought-tolerant rice varieties through accelerated breeding methods and molecular marker-assisted selection has become a global research focus. Drought stress in rice occurs at various developmental stages and physiological levels. During the seedling phase, water shortage hampers germination vigour, shortens coleoptile elongation, inhibits primary root growth, and reduces shoot development, creating early growth disadvantages that build up throughout the plant’s lifecycle [12, 13]. During vegetative growth, drought decreases tillering, leaf area expansion, and photosynthesis, directly limiting biomass production. At the reproductive stage, which is most sensitive to drought, water stress causes spikelet sterility, hampers panicle exsertion, lowers grain filling, and finally severely impacts yield potential [14, 15]. Drought causes cellular impacts such as disrupting osmotic homeostasis, leading to excessive reactive oxygen species (ROS) accumulation, which results in oxidative damage, protein denaturation, membrane destabilization, and metabolic problems [12].

Rice cultivars show significant genetic variation in drought tolerance mechanisms, reflecting the diversity across O. sativa subspecies (indica, japonica, and intermediate types) and the strong selection pressure from traditional and modern breeding efforts [16, 17]. Tolerant cultivars usually feature deep, dense root systems that enhance water uptake from deeper soil layers. They also reduce stomatal conductance to limit water loss through transpiration, improve osmotic adjustment by accumulating compatible solutes, have effective ROS scavenging systems, and preserve the functionality of their photosynthetic machinery under stress [18, 19]. However, the biochemical underpinnings of these tolerance traits—particularly the metabolic reprogramming that enables sustained cellular function under water deficit—remain incompletely understood, especially in cultivars developed outside major Asian breeding programs.

Metabolomics has transformed how we comprehend plant stress responses by offering an unparalleled view of the biochemical phenotype, reflecting the true functional condition of plant metabolism during environmental disturbances [20]. In rice, metabolomic investigations have revealed that drought tolerance involves coordinated modulation of primary metabolism (amino acids, organic acids, sugars) and secondary metabolism (phenylpropanoids, flavonoids, terpenoids), with tolerant genotypes exhibiting distinct metabolic signatures compared to sensitive ones [21, 22]. Gas chromatography-mass spectrometry (GC-MS) offers exceptional capabilities for profiling polar metabolites central to osmotic adjustment, energy metabolism, and stress protection, making it the preferred analytical platform for dissecting drought-responsive biochemical pathways [23, 24]. Previous metabolomic studies in rice have identified several key metabolic responses to drought stress. Accumulation of proline, the most extensively studied osmoprotectant, correlates with improved water retention and membrane stability across diverse rice germplasm [21, 25]. Soluble sugars, including glucose, sucrose, and trehalose, function both as osmolytes and as signaling molecules triggering stress-responsive gene expression [26, 27]. Amino acids beyond proline, especially branched-chain amino acids (leucine, valine, isoleucine), aromatic amino acids (phenylalanine, tryptophan), and glutamate-derived amino acids, accumulate during drought. They function as nitrogen storage forms, biosynthetic precursors, and stress signals [28]. Tricarboxylic acid (TCA) cycle intermediates exhibit complex regulation patterns, with some accumulating to support alternative respiratory pathways and others declining due to photosynthetic inhibition [29–31]. Secondary metabolites, particularly those derived from the shikimate and phenylpropanoid pathways, provide antioxidant protection and structural reinforcement [32].

This study hypothesized that drought-tolerant and drought-sensitive rice cultivars exhibit distinct, tissue-specific metabolic reprogramming patterns in response to water deficit stress, with roots displaying greater metabolic plasticity than leaves due to their role as primary stress sensors. Specifically, we predicted that: (1) tolerant cultivars would demonstrate more extensive metabolic adjustments in root tissues compared to sensitive varieties, (2) cultivar-specific metabolic signatures would correspond to differential drought tolerance capacities, and (3) roots and leaves would employ complementary but distinct metabolic strategies, with roots focusing on stress perception and signaling while leaves implement conserved protective responses. To test these hypotheses, we conducted comprehensive GC-MS-based metabolomic profiling of four Egyptian rice cultivars (Giza 177, Giza 178, Sakha 104, Sakha 108) representing a gradient of drought tolerance phenotypes. Our specific objectives were to: (1) characterize tissue-specific (root vs. leaf) metabolic responses to polyethylene glycol (PEG)-induced drought stress, (2) identify cultivar-specific metabolic signatures that distinguish tolerant from sensitive genotypes, (3) determine the key metabolic biomarkers with highest discriminatory power for drought tolerance assessment, and (4) elucidate the coordinated metabolic pathways that enable root-leaf synergistic stress adaptation. By integrating metabolomic profiles with physiological measurements, this study aims to provide actionable biochemical markers for metabolite-assisted breeding and reveal the mechanistic basis of drought tolerance in Egyptian rice germplasm.

## Materials and methods

### Plant materials and growth conditions

The Pot experiment was conducted in summer 2023. Four Oryza sativa cultivars were obtained from the Rice Research Department in Sakha, Kafr El-Sheikh, Egypt (Table 1). These cultivars are commonly grown in Egypt due to their high yields, distinct morphological traits, and adaptability to diverse climatic conditions. The goal of the study was to assess how drought stress affects these cultivars at the seedling stage. Seeds were surface-sterilized with 3% sodium hypochlorite and germinated on moist blotting paper at around 25 ± 2°c. After pre-germination, seeds from each genotype were planted in plastic trays (30 cm × 20 cm × 10 cm) filled with a 3:1 mix of field soil and farmyard manure. The growth medium was prepared by autoclaving the soil-manure mixture at 121 °C for 15 min and allowing it to cool before use. Seedlings were subsequently transferred to a controlled growth chamber (Conviron CMP6010, Controlled Environments Ltd., Winnipeg, Canada) maintained at 70 ± 5% relative humidity and 24 ± 2 °C day/night temperature with a 16-hour photoperiod under photosynthetic photon flux density of 250–350 µmol m⁻² s⁻¹ provided by LED panels (Philips GreenPower LED Production Module).

**Table 1:** Genetic background and stress tolerance characteristics of rice cultivars used in this study

*N*	Variety	Parentage/pedigree	Type	Stress Tolerance	References	
1	Sakha104	GZ 4096-8-1/GZ 4100-9-1	Japonica	Drought-sensitive		[33]
2	Sakha108	Sakha 101/HR 5824-B-3-2-3//Sakha 101	Japonica	drought- tolerance		[34, 35]
3	Giza177	Giza171/Yomjo No.1//PiNo.4	Indica/Japonica	drought- tolerance		[34, 35]
4	Giza178	Giza 175/Milyang 49	Indica/Japonica	alinity, drought-tolerant		[36]

### Drought stress treatment application and experimental design

Germination rates were recorded after 96 h of incubation. Seeds were deemed germinated if the root length was at least 1 cm and the shoot length was at least 0.5 cm. Initial testing with various PEG6000 concentrations (5%, 10%, 15%, and 20%) indicated that 15% PEG was the most effective for assessing drought tolerance among the cultivars, and it was therefore selected for the drought treatment. Polyethylene glycol (PEG-6000) was selected over water withholding for several scientific reasons PEG induces uniform, reproducible osmotic stress across all plants simultaneously, eliminating spatial heterogeneity inherent in soil-drying experiments [37]; PEG-mediated stress is physiologically equivalent to drought as it creates osmotic potential gradient without causing nutrient imbalances or salt toxicity; PEG allows precise control of stress intensity through concentration adjustment; PEG-induced osmotic stress at the seedling stage is a well-established method for screening drought tolerance in crops [37]; and the controlled nature of PEG treatment enables direct comparison of metabolomic responses across cultivars without confounding environmental variables present in water withholding experiments. This approach is particularly well-suited for metabolomic studies, where reproducibility and standardization are critical.

The experiment employed a randomized complete block design (RCBD) with four replicates. Cultivars (A) included Giza 177, Giza 178, Sekha 104, and Sekha 108, while abiotic treatments (B) consisted of a control without stress and drought stress with 15% PEG applied for 14 days. Each treatment was replicated four times, with each replicate containing 20 seeds. For drought stress induction, 15% (w/v) PEG-6000 solution was prepared by dissolving 150 g of PEG-6000 (molecular weight 6000 Da) in 1 L of distilled water and stirring until complete dissolution. The solution was applied to 14-day-old seedlings by replacing the irrigation water with PEG solution. Each pot received 200 mL of 15% PEG solution, which was maintained at constant volume throughout the 14-day treatment period by adding PEG solution as needed to compensate for evaporation. Control plants received equivalent volumes of distilled water. The PEG solution was prepared fresh every 3 days to maintain consistent osmotic potential and prevent microbial contamination.

### Plant Height, root biomass Measurements, and relative water content

Plant height (PH) was measured from the soil surface to the tip of the longest leaf using a standard ruler and expressed in centimeters.

Root fresh weight (RFW) was recorded at harvest (14 days after PEG treatment) by carefully extracting the plant roots from the growth medium, gently rinsing them with distilled water to eliminate soil particles, blotting off excess water with tissue paper, and immediately weighing them using an analytical balance (± 0.001 g). For dry weight measurement, root samples were oven-dried at 85 °C until their weight remained constant, providing the root dry weight (RDW). Shoot fresh weight (SFW) was measured right after harvest by cutting the shoots at the crown, removing any debris, and weighing them on the same analytical balance. These shoot samples were then oven-dried at 85 °C until reaching a constant weight to determine the shoot dry weight.

Relative water content (RWC) was determined following the method of Barrs and Weatherley [38]. Fresh leaf discs (1 cm²) were weighed immediately after collection (FW), then floated in distilled water for four hours at room temperature to achieve full turgidity before reweighing (TW). Subsequently, discs were oven-dried at 85 °C until constant weight was achieved (DW). RWC was calculated using the formula:

$$\:RWC\:\left(\%\right)\:=\:\frac{(FW\:-\:DW)}{(TW\:-\:DW)}\:\times\:\:100$$

Where: $$FW = Fresh\,weight; TW = Turgid\,weight; DW = Dry\,weight$$ 

The Drought Tolerance Index (DTI) was calculated using the formula: DTI (%) = (FW_drought / FW_control) × 100, where FW_drought represents the fresh weight under 15% PEG treatment, and FW_control represents the fresh weight under control conditions.

### Chlorophyll content

Total chlorophyll index: the degree of green color (measured in SPAD units), determined by a chlorophyll meter (SPAD-502, Minolta Co., Japan). It is represented by the average SPAD value from ten randomly selected leaves in each subplot at 90 DAS, following the described method by Uddling et al. [39].

### Determination of proline content

Proline content was measured using the modified Bates et al. method [40]. After collecting the samples, measure their fresh weight and use about 100 mg for the reaction. Alternatively, snap-freeze the samples in liquid nitrogen and store them at -80 °C if needed. Add 3% sulfosalicylic acid (5 µl/mg of fresh weight) and grind the plant material, keeping the tubes on ice until all samples are processed. Centrifuge the samples at maximum speed for 5 min using a benchtop centrifuge at room temperature. Prepare the reaction mixture separately: 100 µl of 3% sulfosalicylic acid, 200 µl of glacial acetic acid, and 200 µl of acidic ninhydrin. Mix these with 100 µl of the plant extract supernatant. To prevent high pressure and accidental opening, puncture the microcentrifuge tube cap with a needle. Incubate the tubes at 96 °C for 60 min. For extraction, add 1 mL of toluene to the reaction mixture, vortex for 20 s, then leave it to separate for 5 min into the organic and aqueous phases. Handle the tubes with gloves during extraction. Transfer the toluene containing the chromophore to a new tube and measure the absorbance at 520 nm, using toluene as a blank. The concentration of proline can be determined using a standard concentration curve and calculated based on fresh weight (usually expressed as µmole/g of FW). The reaction mixture was measured at 520 nm using a NanoDrop 2000 spectrophotometer (Thermo Scientific, Wilmington, USA) [41].Proline content was calculated per unit as per the formula given below: Proline (µMols) in g^− 1^ FW= (mg proline / ml × ml toluene) / 115.5 mg / µMols) / (g FW / 5) [42].

### Metabolite extraction and GC-MS analysis

Metabolite extraction was performed using a methanol-based protocol optimized for the recovery of polar metabolites. The polar metabolite fraction was selected for analysis based on several considerations: polar primary metabolites (amino acids, organic acids, sugars) function as primary compatible solutes mediating osmotic adjustment, the key physiological mechanism of drought tolerance in rice seedlings [22]; polar metabolites respond rapidly to water deficit (within our 14-day treatment), whereas non-polar metabolites (membrane lipids, cuticular waxes) often represent slower, developmental adaptations; GC-MS with derivatization provides optimal analytical performance for polar compounds, offering superior sensitivity (detection limits 10–100 pmol), reproducibility (CV < 10%), and metabolome coverage (> 150 polar compounds) compared to alternative platforms [21]; focusing on polar metabolites enables direct comparison with the extensive body of drought stress metabolomics literature in rice and other cereals; and polar metabolites represent the metabolic front line of stress response, integrating physiological status with genetic tolerance capacity.

All samples were collected at the same time of day and under identical lighting conditions. Four independent biological replicates were prepared for each cultivar × treatment combination (4 cultivars × 2 treatments × 4 replicates = 32 samples total). Frozen tissue samples (100 mg) were ground to fine powder using liquid nitrogen and extracted with 500 µL of ice-cold 80% (v/v) methanol containing 0.1% formic acid. Samples were vortexed for 30 s and then incubated on ice for 5 min. This was followed by centrifugation at 12,000× g for 20 min at 4 °C. The supernatant was carefully transferred to a clean microcentrifuge tube and diluted with ultrapure water (Milli-Q, Millipore, Bedford, MA, USA) to achieve 53% methanol content. The diluted extract was subjected to a second centrifugation step (12,000× g for 10 min at 4 °C), and the resulting supernatant was used for GC-MS analysis. Gas chromatography-mass spectrometry analysis was conducted using an Agilent 7890B gas chromatograph coupled with a 5977 A mass selective detector (Agilent Technologies, Santa Clara, CA, USA). Chromatographic separation was achieved using a Hypersil Gold C18 column (150 mm × 2.1 mm, 1.9 μm particle size; Thermo Fisher Scientific, Waltham, MA, USA) maintained at an initial temperature of 40 °C. The oven temperature program consisted of an initial hold at 40 °C for 2 min, followed by a linear ramp at 10 °C min⁻¹ to 320 °C with a final hold of 5 min, providing a total run time of 35 min per sample. The injection port temperature was set to 250 °C in splitless injection mode with a 1 µL injection volume. Helium (99.999% purity) was used as carrier gas at a constant flow rate of 1.2 mL/min⁻. Mass spectrometry was performed in electron ionization (EI) mode at 70 eV with an ion source temperature of 230 °C and a quadrupole temperature of 150 °C. The mass spectrometer was operated in scan mode with a mass range of 50–600 m/z and a scan rate of 3.5 scans per second. The transfer line temperature was maintained at 280 °C. System performance was monitored by injecting quality control samples (pooled biological samples) after every 10 sample injections. Blank injections were performed between sample batches to monitor system contamination.

#### Metabolite identification and data processing

Metabolite identification was achieved by comparing with the NIST 17 mass spectral library (National Institute of Standards and Technology, Gaithersburg, MD, USA) using Agilent MassHunter Qualitative Analysis software (Version B.08.00). Identification criteria included a minimum spectral match factor of 80% and a retention time deviation of less than 5% compared to authentic standards when available. Peak integration was performed using Agilent MassHunter Quantitative Analysis software (Version B.09.00), with manual verification of integration boundaries. Quality control measures involved the use of ribitol (Sigma-Aldrich) as an internal standard at a final concentration of 10 µg/mL to normalize extraction efficiency and instrument response variations. Raw data preprocessing involved filtering compounds based on peak-intensity thresholds (minimum of 1000 counts) and on their presence in at least 80% of samples within each treatment group. Missing values were addressed using K-nearest neighbor (KNN) imputation for metabolites with less than 20% missing data across all samples. In contrast, metabolites exceeding this threshold were removed from the analysis. Data normalization was done through log₂ transformation, followed by auto-scaling (mean-centring and unit variance scaling) to account for different metabolite concentration ranges and ensure equal weight in multivariate analyses [43].This comprehensive protocol follows best practices in GC-MS metabolomics [43, 44], and ensures reproducible, high-quality data suitable for comparative metabolomics.”

### Statistical analysis

Statistical analyses were performed using MetaboAnalyst 5.0 (https://www.metaboanalyst.ca/, accessed on March 20, 2025) and R software (Version 4 3 4.3.0, R Foundation for Statistical Computing, Vienna, Austria). Morpho-physiological data were subjected to two-way analysis of variance (ANOVA) with cultivar and treatment as fixed factors, and biological replicates were treated as random effects. Normality and homoscedasticity assumptions were verified using the Shapiro-Wilk and Levene’s tests, respectively. When significant differences were found, means were compared using Tukey’s honestly significant difference (HSD) test at α = 0.05. For metabolomic data, both univariate and multivariate statistical analyses were applied. Univariate analysis involved unpaired t-tests for pairwise comparisons between treatments within each cultivar, with multiple testing corrections using the Benjamini-Hochberg false discovery rate (FDR) procedure to reduce Type I errors. Significance was defined as FDR-adjusted *p* < 0.05. Effect sizes were measured using Cohen’s d for all significant metabolite differences, with 95% confidence intervals calculated via bootstrap resampling (*n* = 1000). Multivariate analyses included hierarchical cluster analysis (HCA), principal component analysis (PCA), and partial least squares discriminant analysis (PLS-DA) (figure S1 and S2). PCA was conducted on mean-centred and unit-variance-scale data to identify key sources of variation in metabolomic profiles. PLS-DA provided supervised classification of treatment groups, with model validation through 10-fold cross-validation and permutation testing (*n* = 500). Variables with the highest discriminative power were identified using variable importance in projection (VIP) scores; VIP > 1.6, combined with FDR *p* < 0.01, served as biomarker criteria. Hierarchical clustering was performed using Euclidean distance and Ward’s linkage method to reveal clustering patterns among samples and metabolites. Bootstrap validation (*n* = 1000) was used to assess the stability of multivariate models, ensuring reliable interpretation. Post-hoc power analysis confirmed that all significant differences had a statistical power greater than 0. 0.8 for detecting meaningful biological effects. Data are shown as means ± standard error of the mean (SEM) unless otherwise noted. All statistical tests used a significance threshold of *p* < 0.05. Experimental procedures complied with institutional guidelines and regulations for plant research.

## Results

### Morpho-Physiological responses to drought stress

**Fig. 1:**
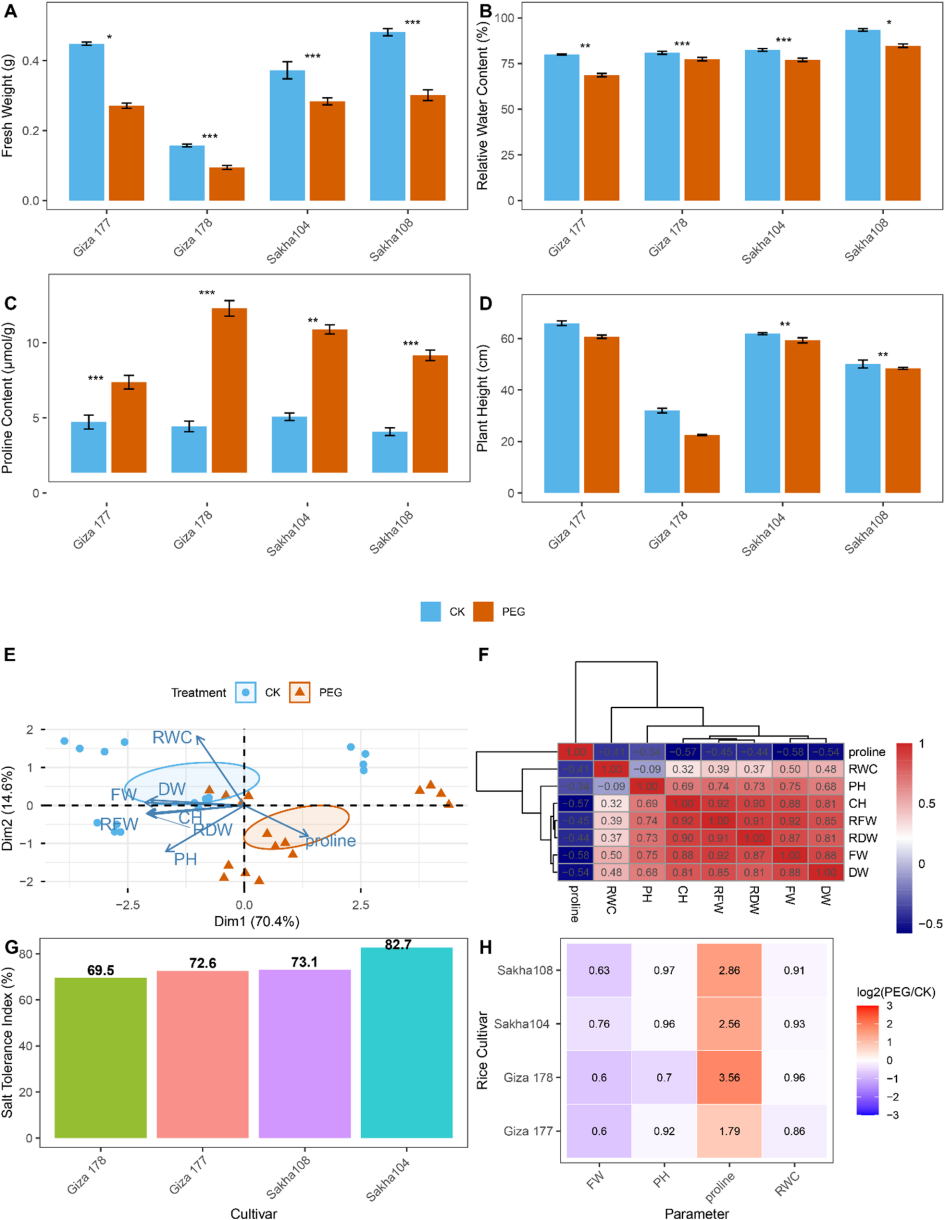
Differential physiological responses of rice cultivars to drought stress. **A** Fresh weight of rice seedlings under control conditions (CK, blue bars) and drought stress (15% PEG, orange bars). **B** Leaves’ relative water content (RWC) in response to drought. **C** Proline levels in leaves under control and drought. **D** Plant height indicating growth reduction during drought. Data in panels A-D are shown as means ± SE (*n* = 4 biological replicates). Significance between control and drought within each cultivar is marked by horizontal brackets with asterisks: * *p* < 0.05; ** *p* < 0.01; *** *p* < 0.001. **E** PCA biplot displaying separation between control (blue circles) and drought-treated (orange triangles) samples across cultivars. Blue arrows indicate loadings for physiological variables: FW (fresh weight), DW (dry weight), RFW (relative fresh weight), RDW (relative dry weight), RWC (relative water content), CH (chlorophyll), PH (plant height). **F** Correlation matrix heatmap showing Pearson coefficients from − 0.5 (blue, negative) to 1.0 (red, positive). Hierarchical clustering dendrogram illustrates parameter relationships. **G** Drought tolerance indices are calculated as percent of fresh weight retained under stress compared to control. The numbers above bars display these percentages. **H** Heatmap of log₂ PEG/CK ratios for key parameters (FW, PH, proline, RWC) across four cultivars. Red indicates upregulation; blue indicates downregulation; white signifies no change. Dendrogram on the left groups cultivars by overall response similarity

Drought stress induced by 15% PEG treatment for 14 days caused significant physiological and morphological changes across all four rice cultivars, revealing differential tolerance capacities (Fig. 1). Fresh weight decreased significantly under drought stress in all cultivars, with reductions ranging from 24% to 44% (Fig. 1A). Giza 177 and Sakha 108 showed the largest declines (44% and 41%, respectively), while Sakha 104 exhibited the smallest reduction (24%). Despite these decreases, Sakha 108 and Sakha 104 maintained higher absolute biomass under stress compared to the Giza cultivars. Plant height similarly declined under drought, though responses varied by cultivar (Fig. 1D). Giza 178 experienced the most severe reduction (33%), while Giza 177 and Sakha 104 showed minimal height changes (6–8%). These differential responses reflect cultivar-specific strategies for balancing growth and stress defence. Relative water content (RWC) declined in all cultivars under drought stress, with reductions ranging from 6% to 15% (Fig. 1B). Giza 177 showed the largest decrease (15%), while Giza 178 maintained the most stable RWC (6% decline). Notably, Sakha 108 maintained the highest absolute RWC under both control and drought conditions, with a moderate 9% reduction, correlating strongly with its superior drought tolerance. Proline content increased dramatically across all cultivars, demonstrating its central role in osmotic adjustment (Fig. 1C). The magnitude of accumulation varied widely: Giza 178 showed the highest increase (3.9-fold), followed by Sakha 108 (3.1-fold), Sakha 104 (2.7-fold), and Giza 177 (1.9-fold). However, the relationship between proline levels and overall tolerance was complex: Sakha 108 maintained superior physiological performance despite moderate proline accumulation, suggesting the presence of multiple complementary tolerance mechanisms.

Principal component analysis (PCA) clearly separated control and drought-treated samples, with the first two components explaining 85% of total variation (PC1: 70.4%, PC2: 14.6%) (Fig. 1E). Control samples clustered with higher biomass and water content, whereas drought samples were strongly associated with elevated proline levels. Sakha 108 and Sakha 104 occupied intermediate positions, suggesting more effective stress mitigation. Correlation analysis revealed strong positive relationships between proline and biomass parameters (*r* = 0.92 with relative dry weight), and among water-related measurements (Fig. 1F). These patterns demonstrate coordinated physiological adjustments where declining water status triggers compensatory osmolyte accumulation. Drought tolerance indices based on biomass retention ranked the cultivars as follows: Sakha 104 (82.7%) > Sakha 108 (73.1%) > Giza 177 (72.6%) > Giza 178 (69.5%) (Fig. 1G). Heatmap analysis of log₂-transformed stress/control ratios confirmed proline as the most responsive parameter across all cultivars, with fold-changes ranging from 3.5× to 11.8× (Fig. 1H). Hierarchical clustering grouped Sakha 104 and Sakha 108 together, distinct from the more stress-sensitive Giza cultivars.

### Multivariate analysis of GC-MS metabolomic data reveals Tissue-Specific and Cultivar-Dependent responses

#### Leaf tissue metabolomic responses

**Fig. 2:**
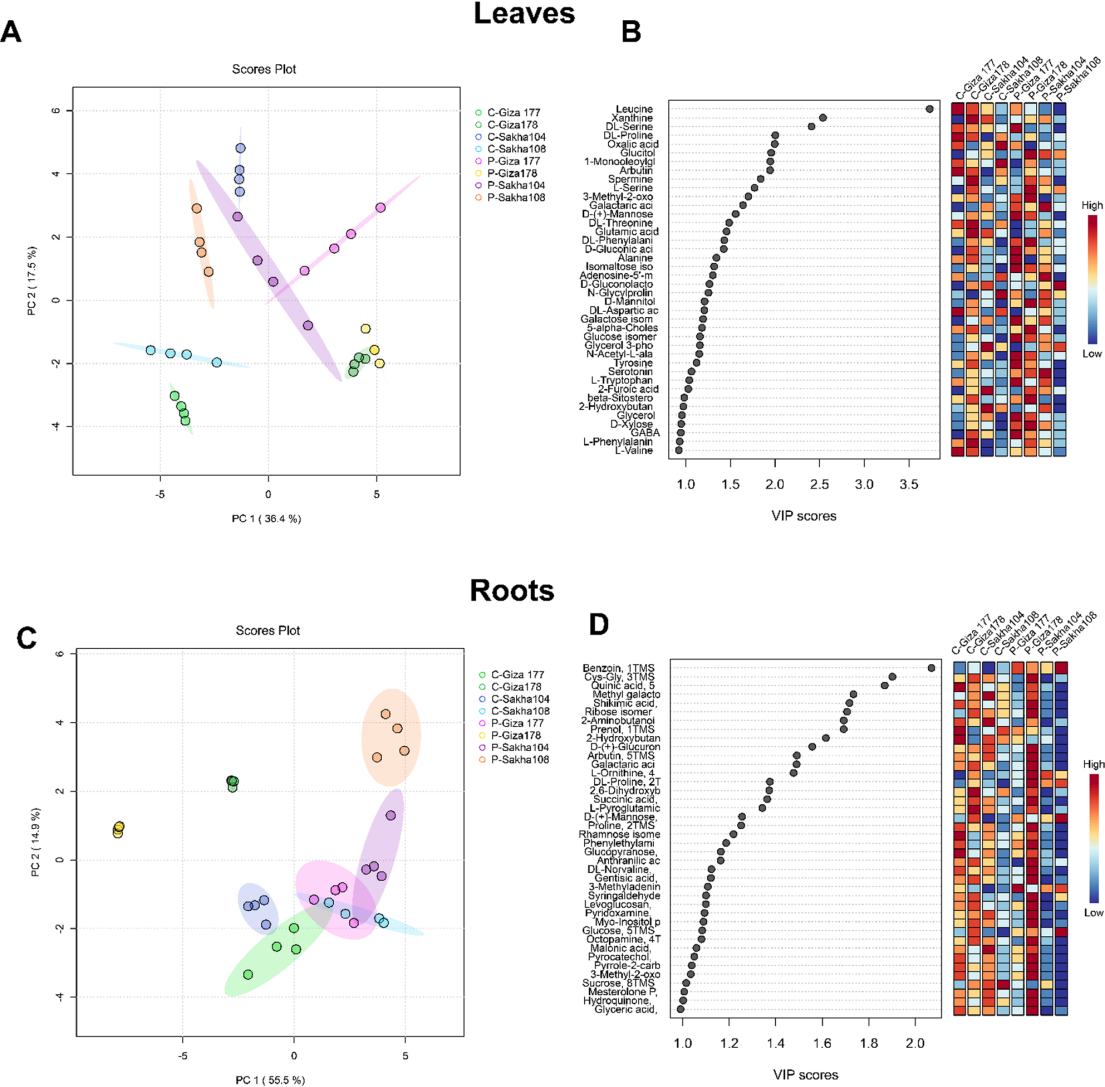
Principal Component Analysis (PCA) and Variable Importance in Projection (VIP) analysis of metabolomic profiles from four rice cultivars under control and drought tress conditions based on GC-MS data. **A** PCA score plot of leaf tissue metabolites with PC1 explaining 36.4% and PC2 accounting for 17.5% of total variance. **B** VIP score plot for leaf samples, illustrating metabolite importance rankings based on weighted sums of squares of PLS loadings and explained variance contributions. **C** PCA score plot of root tissue metabolites with PC1 explaining 55.4% and PC2 accounting for 14.9% of total variance. **D** VIP score plot for root samples, displaying the relative importance of individual metabolites in distinguishing treatment groups and cultivar responses

PCA of GC-MS metabolomic data revealed moderate metabolic differentiation between control and drought-treated leaf samples, with the first two principal components explaining 53.9% of the total variance (PC1: 36.4%, PC2: 17.5%) (Fig. 2A). Clear clustering by cultivar and treatment was observed, with drought-treated samples generally separated from controls along PC1. Notable overlaps occurred between Sakha 104 and Giza 177 under drought stress, and between Sakha 108 and Giza 177 under control conditions, suggesting shared metabolic features and potential convergence in stress responses. The moderate separation indicates that while drought significantly influences foliar metabolism, conserved metabolic programs are maintained across cultivars.

#### Root tissue metabolomic responses

Root tissue analysis revealed substantially clearer metabolic differentiation than leaves, with the first two principal components explaining 70.4% of the total variance (PC1: 55.6%, PC2: 14.8%) (Fig. 2C). The nearly two-fold higher variance explained by PC1 in roots (55.6%) compared to leaves (36.4%) indicates that root metabolomes undergo more extensive reorganization in response to drought. Cultivar-treatment groups formed more distinct clusters in roots, with Giza 178 exhibiting the most pronounced metabolic differentiation under both control and drought conditions, suggesting unique constitutive and stress-responsive metabolic features. The enhanced metabolic plasticity in roots supports their role as primary stress sensors undergoing more extensive reprogramming than aerial tissues.

#### Variable importance in projection (VIP) analysis

VIP analysis identified 40 metabolites with scores > 1.0 that contribute significantly to treatment discrimination in both leaf and root tissues (Fig. 2B and D). In leaves, top discriminatory metabolites (VIP > 2.5) included leucine, DL-serine, and various organic acids. Mid-tier metabolites (VIP 2.0-2.5) encompassed amino acids (alanine, valine, proline), sugar derivatives, and TCA cycle intermediates. Root VIP analysis revealed a partially overlapping but distinct metabolite set, with benzoin, ribose isomers, and amino acid derivatives showing highest discriminatory power. Using a stringent threshold of VIP > 1.6, 13 key biomarkers were identified that strongly differentiate drought responses across cultivars, representing amino acid metabolism, TCA cycle, sugar metabolism, and secondary metabolite pathways. The total dataset comprised 114 polar metabolites in leaves and 97 in roots, with roots showing more coordinated metabolic responses despite lower absolute metabolite diversity. While we emphasize the 13 high-priority biomarkers with VIP > 1.6 for their superior discriminatory power and potential breeding applications (Table 2), the additional 27 metabolites in the VIP 1.0-1.6 range also contribute significantly to cultivar differentiation (Supplementary Table S1-S4). These moderate-VIP metabolites include secondary amino acids (glycine, threonine, asparagine), additional TCA cycle intermediates (fumarate, malate), and sugar derivatives (glucose-6-phosphate, fructose-6-phosphate), which collectively enhance the metabolic signature of drought response. The dual-threshold approach allows us to identify both high-confidence biomarkers (VIP > 1.6) for immediate breeding applications and supporting metabolites (VIP 1.0-1.6) for comprehensive mechanistic understanding.

**Table 2:** High-priority metabolic biomarkers (VIP > 1.6) for drought tolerance screening in rice breeding programs

Metabolite	VIP Score (Comp. 1)	Tissue	Primary Pathway	Fold-Change (Drought/Control)	Breeding Application
Leucine	3.73	Leaf	BCAA metabolism	2.3–3.1×	Energy homeostasis marker
Xanthine	2.54	Leaf	Purine metabolism	1.8–2.4×	Nucleotide turnover indicator
DL-Serine	2.41	Leaf	Amino acid metabolism	1.9–2.6×	One-carbon metabolism marker
Proline	2.00	Leaf	Amino acid metabolism	1.9–3.9×	Primary screening marker
Oxalic acid	2.00	Leaf	Organic acid metabolism	2.0–3.0×	Stress intensity indicator
Spermine	1.84	Leaf	Polyamine biosynthesis	2.8×	Membrane protection capacity
Benzoin	2.07	Root	Aromatic metabolism	1.6–2.2×	Secondary metabolism activity
Cys-Gly	1.90	Root	Glutathione metabolism	2.1–2.8×	Antioxidant capacity
Quinic acid	1.87	Root	Shikimate pathway	2.4×	Phenolic biosynthesis potential
Shikimic acid	1.72	Root	Shikimate pathway	2.6×	Aromatic compound biosynthesis
Ribose	1.71	Root	Pentose phosphate pathway	2.3×	Redox homeostasis marker
Malic acid derivative	1.62	Root	TCA cycle	2.5×	Energy metabolism status

### Cultivar-Specific metabolic responses to drought stress

#### Hierarchical clustering and comparative analysis

**Fig. 3:**
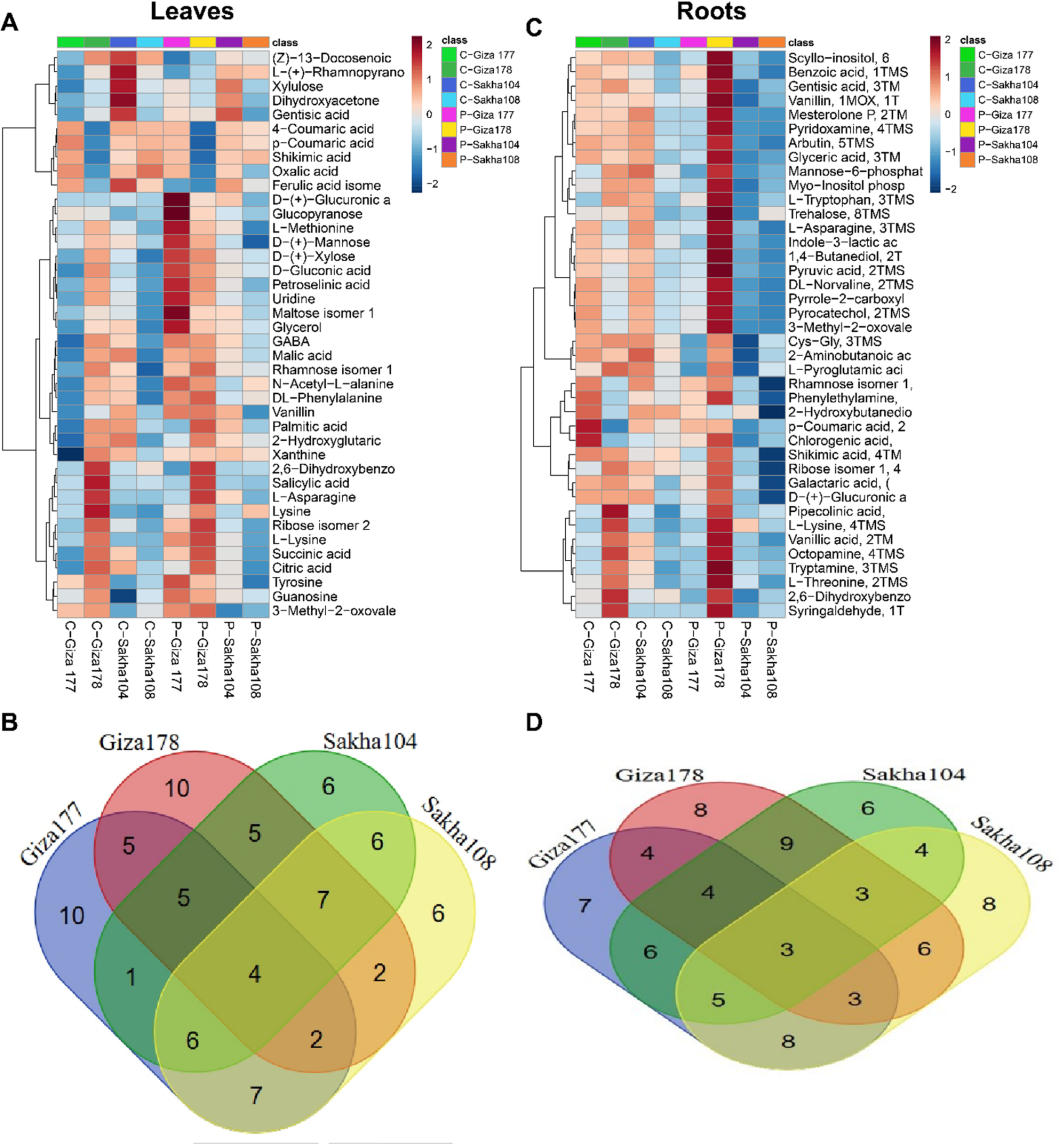
Metabolomic profiling and comparative analysis of drought stress responses across four rice cultivars. Heatmaps display hierarchical clustering of the top 40 most variable metabolites by VIP score, with experimental conditions arranged in rows and metabolite profiles in columns. Color intensity represents relative metabolite concentrations, with red indicating elevated abundance and blue denoting reduced levels. **A** Leaf tissue metabolomic heatmap analysis. **B** Venn diagram illustrating shared and unique metabolites among cultivars at the leaf level. **C** Root tissue metabolomic heatmap analysis. **D** Venn diagram showing metabolite distribution patterns among cultivars at the root level

Hierarchical clustering of metabolomic profiles revealed distinct tissue-specific responses to drought stress at both leaf and root levels. The heatmap was designed with a matrix layout, showing rows as individual metabolites and columns representing experimental conditions (control versus drought-treated samples) across different cultivars. Metabolite levels were visualized using a color intensity scale: red for high abundance, white/orange for intermediate levels, and blue for lower concentrations compared to the dataset average. Analyzing the top 40 VIP-selected metabolites in the heatmap clarified the concentration changes across treatments. Leaf responses are shown in Fig. 3A, and root responses in Fig. 3C. This approach demonstrated tissue-specific and cultivar-dependent metabolic reprogramming under drought conditions, emphasizing the complex mechanisms involved in plant stress adaptation.

#### Leaf tissue metabolite profiling and Cultivar-Specific signatures

The leaf tissue heatmap (Fig. 3A) showed distinct metabolic signatures for each cultivar under both control and drought conditions. Hierarchical clustering of metabolites grouped compounds with similar response patterns, forming clear blocks in the heatmap that indicate coordinated metabolic pathways. Several metabolites were universally upregulated across all cultivars during drought stress, appearing as vertical red bands in the drought-treated columns. These included compounds such as proline, certain amino acids, and sugar derivatives, consistent with their known roles as osmoprotectants and compatible solutes. Conversely, some metabolites consistently decreased under drought stress across cultivars, shown as blue bands in the stress treatment columns. These typically included compounds linked to active growth and primary metabolism, reflecting a shift from growth to survival under stress. Cultivar-specific responses were also evident, with certain metabolites accumulating more in specific cultivars than others. For example, leucine and DL-serine showed particularly high levels in drought-stressed Giza 177 samples (intense red coloration), while other cultivars displayed more moderate increases. The clustering patterns revealed major groups: (1) amino acids and nitrogen compounds forming a coherent cluster, (2) organic acids, including TCA cycle intermediates, (3) sugars and derivatives, and (4) secondary metabolites like phenolic compounds. The consistent clustering of these metabolite classes across samples supports the biological relevance of the analytical approach and shows coordinated regulation within metabolic pathways.

#### Root tissue metabolite profiling reveals enhanced metabolic plasticity

The root tissue heatmap (Fig. 3C) showed more pronounced metabolic differences compared to leaf tissues, aligning with the PCA results that indicate greater variability in root metabolic profiles. The color intensity changes were more extreme in roots, with more prominent red and blue areas indicating larger fold-changes in metabolite levels under drought stress. This increased metabolic flexibility in roots reflects their role as primary stress sensors and the first point of drought detection. Several metabolites showed distinct cultivar-specific patterns of accumulation in roots. Giza 177 exhibited unique upregulation of 3-methyladenine and certain organic acids under drought conditions, appearing as isolated red blocks in the Giza 177 drought column. Giza 178 displayed significant accumulation of Cys-Gly, shikimic acid, ribose isomers, galactaric acid, and L-ornithine under drought stress, creating a characteristic metabolic signature that sets it apart from other cultivars. Sakha 104 showed strong upregulation of Adenosine-5-monophosphate and DL-Aspartic acid, while Sakha 108 demonstrated a preference for D-gluconolactone and glycerol 3-phosphate accumulation. The hierarchical clustering of metabolites in roots revealed both conserved and divergent response patterns. A subset of metabolites was consistently upregulated across all cultivars (a universal stress response), while another significant group displayed cultivar-specific regulation patterns (adaptive diversity). The latter is particularly valuable for pinpointing genetic factors underlying drought tolerance, as these metabolites may represent cultivar-specific adaptations that influence stress resilience. Notably, the heatmap also showed that some metabolite classes had tissue-specific regulation patterns. For example, certain amino acids accumulated strongly in leaves but showed modest changes in roots, whereas some organic acids and secondary metabolites exhibited the opposite trend. This tissue-specific metabolic specialization suggests that leaves and roots adopt complementary strategies for drought adaptation, with leaves focusing on osmotic regulation and photoprotection, and roots emphasizing stress signaling and resource mobilization.

#### Venn diagram analysis of shared and unique metabolites

Venn diagram analysis of the 114 leaf metabolites revealed only four universally expressed compounds across all cultivars (Fig. 3B), indicating limited metabolic conservation despite shared drought exposure. Each cultivar exhibited 6–10 unique metabolites, with Giza 177 and Sakha 104 sharing the most pairwise overlap (10 metabolites). The predominance of cultivar-specific metabolites over the small universal core reflects substantial metabolic diversity in leaf drought responses.

#### Root metabolite distribution across cultivars

Root metabolomic analysis identified merely three universally expressed metabolites across all cultivars (Fig. 3D), representing an even smaller core than leaves (4 metabolites) and confirming greater genotype-dependent metabolic plasticity in roots. Each cultivar possessed 6–8 unique root metabolites, with Giza 178 and Sakha 104 sharing the highest pairwise overlap (9 metabolites). This pattern indicates roots deploy diverse, cultivar-specific metabolic strategies, providing rich genetic variation for breeding-assisted selection.

### Hierarchical clustering analysis of rice cultivar metabolomic profiles

**Fig. 4:**
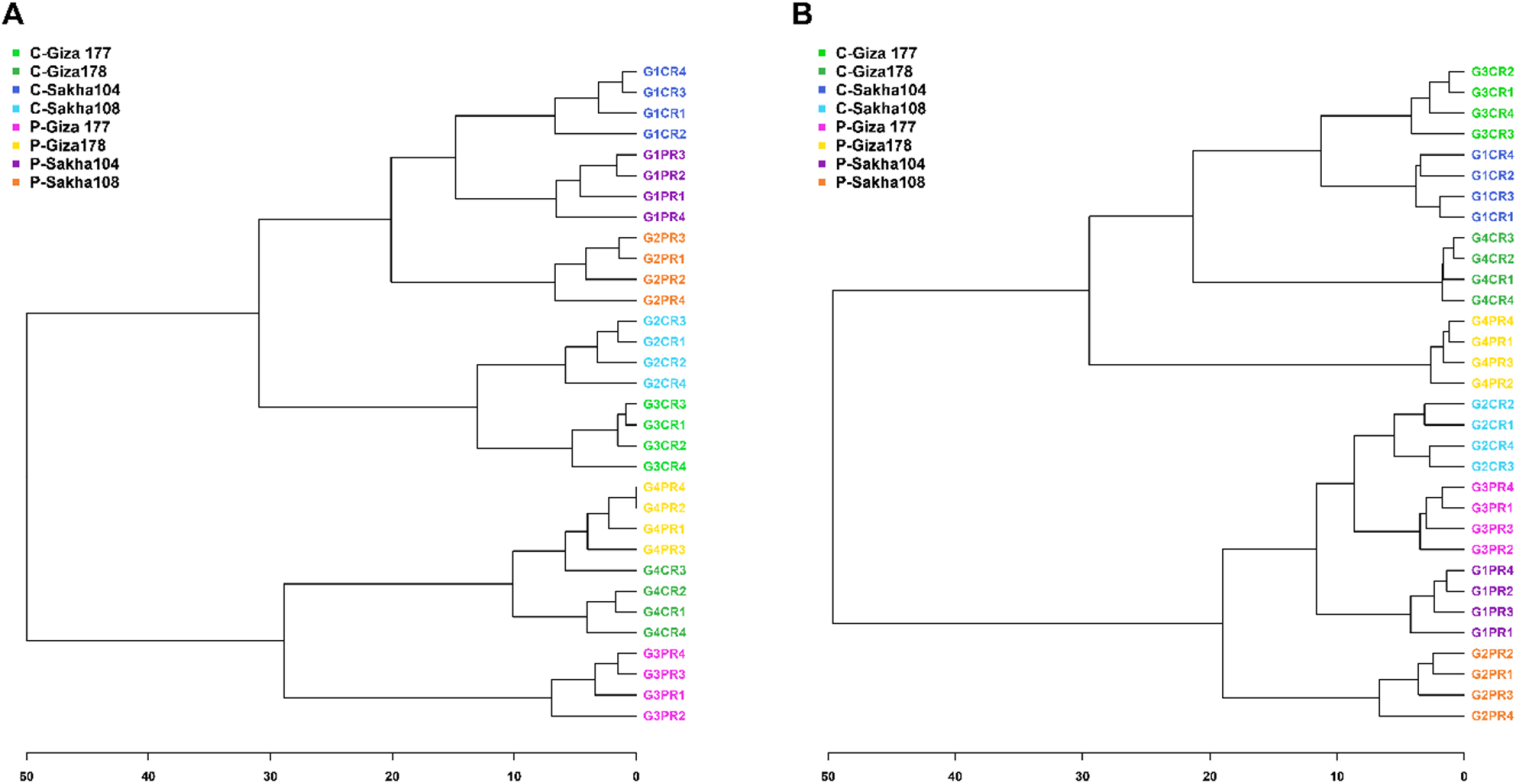
Hierarchical cluster analysis of metabolomic profiles in rice cultivars under drought stress conditions. Dendrograms showing hierarchical clustering of metabolomic profiles from (**A**) leaf tissues and (**B**) root tissues of four rice cultivars (Giza 177, Giza 178, Sakha 104, Sakha 108) under control (C-) and drought stress (PEG-) conditions. Color coding represents different cultivars: green (Giza 177), blue (Giza 178), pink (Sakha 104), and orange (Sakha 108). Numbers following cultivar names indicate biological replicates (1–4). Clustering was performed using Euclidean distance and Ward’s linkage method based on normalized metabolite intensities

Hierarchical clustering revealed distinct tissue-specific patterns in metabolomic responses to drought stress (Fig. 4A-B).

#### Leaf tissue clustering patterns

Leaf metabolomic profiles clustered primarily by treatment rather than by cultivar (Fig. 4A). Control and drought-stressed samples formed two major branches, indicating that drought stress imposes a dominant, conserved metabolic signature across genotypes in leaf tissues. Within the drought cluster, Giza 177 and Sakha 104 grouped together, suggesting similar foliar stress responses despite different genetic backgrounds. Sakha 108 formed a distinct sub-cluster within the drought group, reflecting its unique metabolic profile. Control samples showed moderate cultivar-specific variation but remained more similar to each other than to any drought-treated samples. The treatment-driven clustering pattern in leaves indicates that foliar metabolism responds to drought through largely conserved mechanisms, with genotypic variation playing a secondary role. This finding aligns with the moderate variance explained by principal components in leaf PCA analysis (53.9%).

#### Root tissue clustering patterns

Root metabolomic profiles exhibited more complex clustering patterns with greater variability between cultivars and less distinct separation based solely on treatment (Fig. 4B). Unlike leaves, root clustering was influenced by both cultivar identity and treatment status, with responses specific to each cultivar being more evident. Sakha 104 and Sakha 108 samples formed main branches that included both control and drought-treated replicates, indicating strong genotypic influences on root metabolism. Giza 177 and Giza 178 similarly clustered primarily by cultivar, then by treatment. The cultivar-driven clustering observed in roots suggests higher metabolic plasticity and genotype-specific adaptation strategies compared to leaves. This pattern aligns with the higher variance explained in root PCA (70.4%), indicating that roots display more pronounced and diverse metabolic responses to drought. Sakha 108 exhibited particularly distinct clustering patterns, consistent with its high drought-tolerance index (73.1%), while Giza cultivars showed more variable responses.

#### Tissue-Specific metabolic strategies

The contrasting clustering patterns between tissues reveal fundamentally different stress adaptation strategies. Leaves employ relatively uniform stress responses dominated by treatment effects, suggesting conserved defense mechanisms focused on osmotic adjustment and photoprotection. Roots, as primary stress sensors in direct contact with the soil environment, deploy more diverse, cultivar-specific metabolic strategies that likely reflect genetic differences in stress perception, signaling, and resource allocation. These findings validate the tissue-specific nature of drought adaptation and support the conclusion that root metabolic diversity underlies cultivar-dependent tolerance mechanisms. The greater metabolic flexibility in roots provides a broader substrate for natural selection and targeted breeding to enhance drought resilience.

### Volcano plot analysis of differential metabolite expression under drought stress

**Fig. 5:**
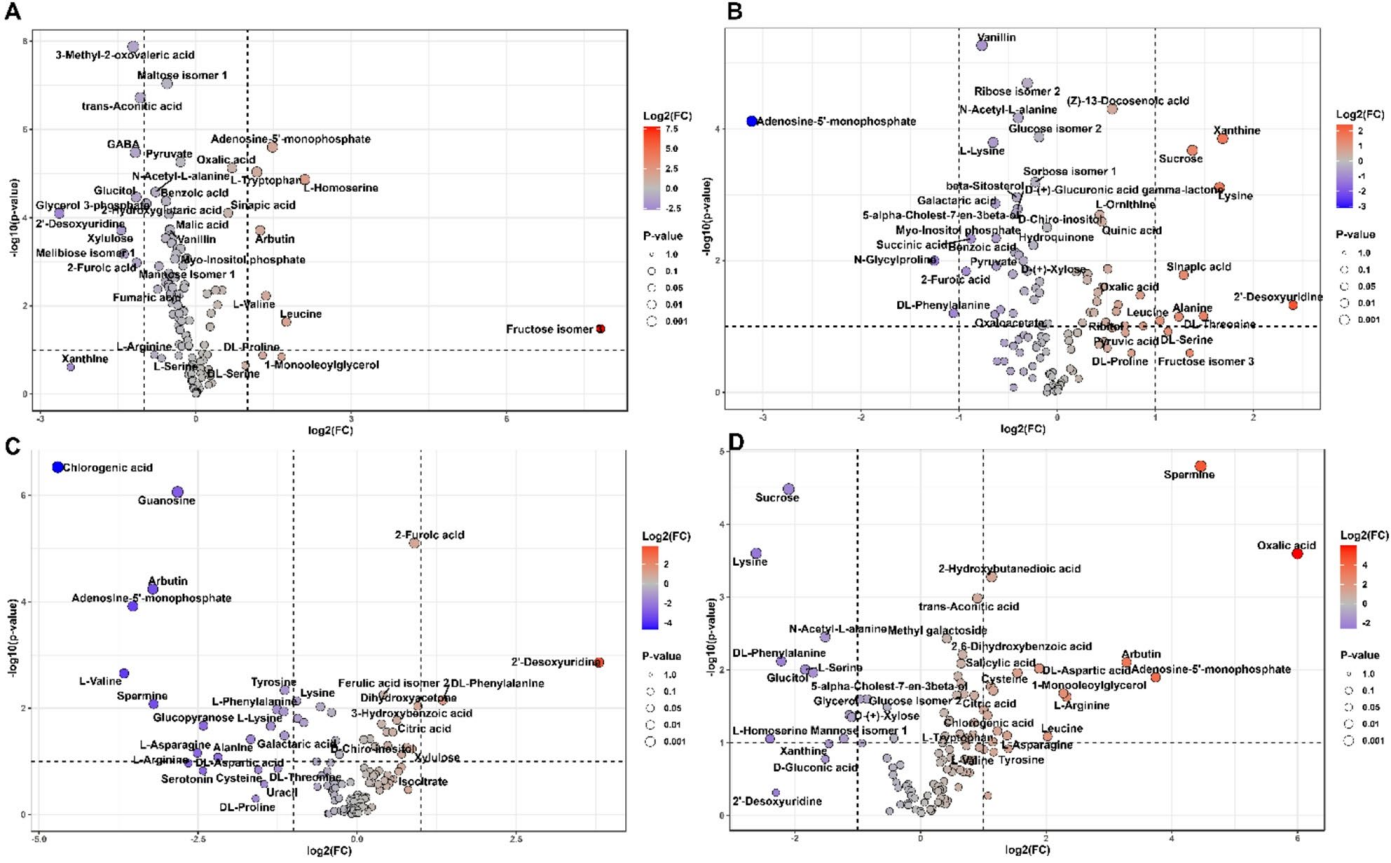
Leaf tissue metabolomic responses to drought stress across rice cultivars. Volcano plots displaying differential metabolite accumulation in leaf tissues of (**A**) Giza 177, (**B**) Giza 178, (**C**) Sakha 104, and (**D**) Sakha 108 following 14-day exposure to PEG treatment. X-axis shows fold change relative to control conditions; Y-axis shows statistical significance. Color gradient represents fold-change magnitude: red indicates upregulation, blue indicates downregulation. Metabolite names are labeled for compounds exceeding significance thresholds. Point sizes correspond to significance levels

**Fig. 6:**
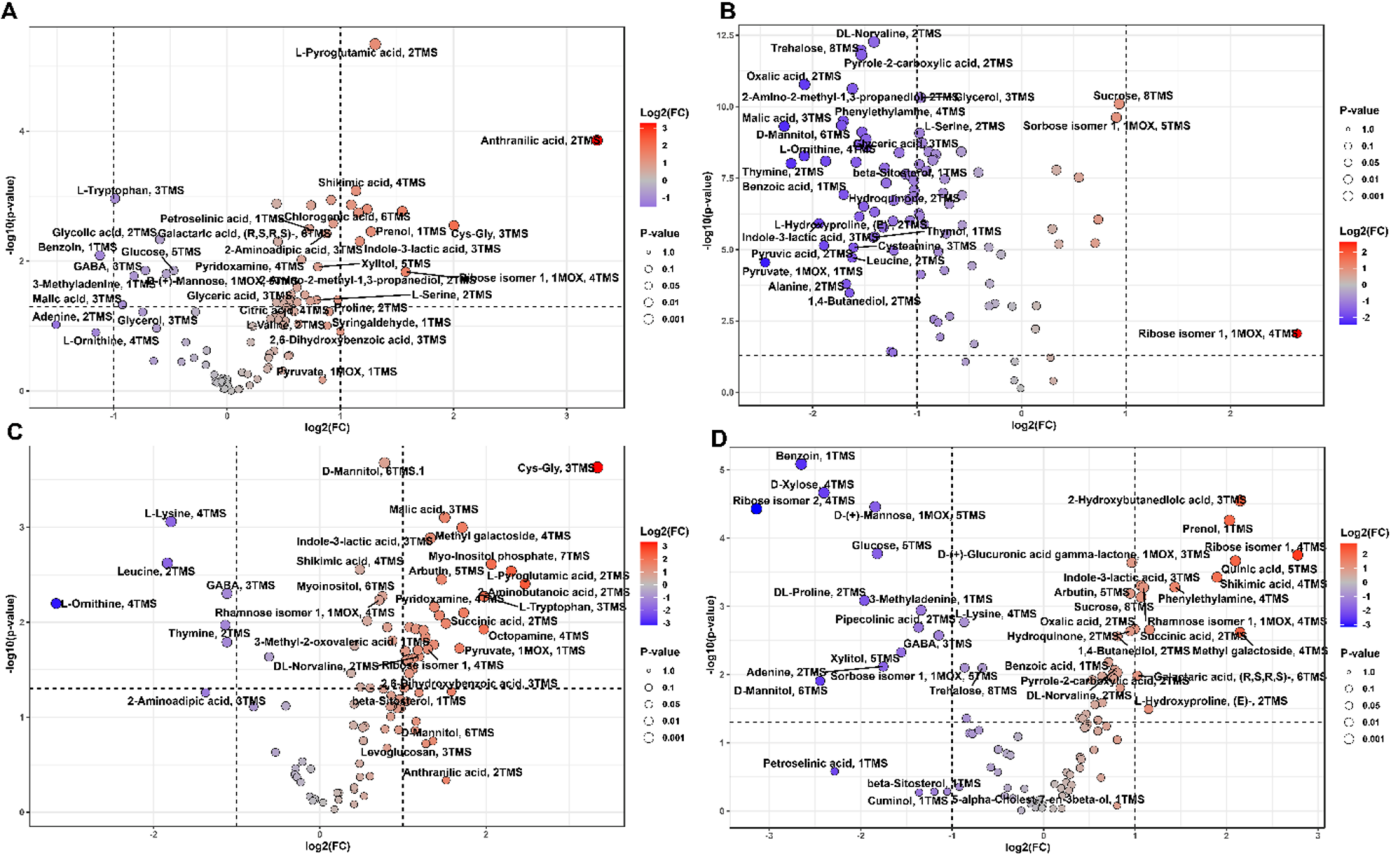
Root tissue metabolomic responses to drought stress across rice cultivars. Volcano plots displaying differential metabolite accumulation in root tissues of (**A**) Giza 177, (**B**) Giza 178, (**C**) Sakha 104, and (**D**) Sakha 108 following 14-day exposure to PEG treatment. X-axis shows fold change relative to control conditions; Y-axis shows statistical significance. Color gradient represents fold-change magnitude: red indicates upregulation, blue indicates downregulation. Metabolite names are labelled for compounds exceeding significance thresholds. Point sizes correspond to significance levels. The volcano plot analyses reveal drought stress induces extensive metabolic reprogramming in both leaf and root tissues, with cultivar- and tissue-specific patterns. The magnitude and direction of metabolite changes vary among cultivars, reflecting genetic differences in drought tolerance. Root responses are more pronounced and diverse than leaves, highlighting their role as primary stress sensors. Consistently upregulated metabolites like oxalic acid, shikimic acid, and certain sugars across cultivars, alongside specific responses, offer insights into drought tolerance mechanisms and potential targets for rice stress resilience improvement

Volcano plot analysis identified metabolites with significant differential accumulation under drought, illustrating cultivar-specific responses (Figs. 5 and 6). The plots display fold-change on the x-axis and significance on the y-axis. Metabolites surpassing significance thresholds are labelled, with red indicating upregulation, blue showing downregulation, and grey representing non-significant changes. Point sizes reflect statistical significance, with larger points indicating greater significance.

#### Leaf tissue metabolomic response to drought stress

Giza 177 showed moderate metabolic changes under drought stress, with significant increases in amino acids, such as L-tryptophan, and in organic acids, such as oxalic acid (Fig. 5A). Metabolites like 3-Methyl-2-oxovaleric acid, maltose isomer, xanthine, and L-Arginine were downregulated, indicating decreased carbohydrate and purine metabolism. Giza 178 exhibited a broader response, with increased levels of amino acids such as Alanine, Leucine, DL-Proline, and Lysine, as well as upregulated organic acids such as oxalic acid (Fig. 5B). Some metabolites, including Vanillin and succinic acid, were downregulated, suggesting complex regulation of metabolic pathways. Sakha 104 displayed a unique profile with moderate changes, but notable increases in organic acids, such as citric acid and isocitrate, and secondary metabolites, such as ferulic acid, highlighting cultivar-specific responses (Fig. 5C). Downregulated compounds included Chlorogenic acid, Guanosine, Valine, Arbutin, and DL-Proline, indicating complex regulation and different osmotic strategies. Sakha 108 exhibited the most significant metabolic changes, with substantial upregulation of oxalic acid, spermine, adenosine-5’-monophosphate, arbutin, and DL-aspartic acid, and downregulation of sucrose, lysine, N-Acetyl-L-alanine, and 2’-deoxyuridine (Fig. 5D). This cultivar demonstrates extensive metabolic reprogramming under drought, likely enhancing its tolerance.

#### Root tissue metabolomic response to drought stress

Giza 177 roots showed moderate metabolic shifts, with key organic acids, amino acids, and intermediates upregulated, indicating active phenylpropanoid and amino acid pathways, while some compounds like tryptophan and glucose were downregulated, possibly due to conversion or increased utilization (Fig. 6A). Giza 178 roots displayed specific sugar compound upregulation, suggestive of carbohydrate metabolism adjustments for osmoprotection, while some amino acids decreased, reflecting targeted regulation (Fig. 6B). Sakha 104 roots exhibited the most extensive metabolic changes under drought, with many compounds, including GSH-related dipeptides, malic acid, and sugar derivatives, upregulated for stress response; others like amino acids and GABA were downregulated, indicating metabolic reallocation, with tissue-specific adjustments across cultivars (Fig. 6C). Sakha 108 roots demonstrated a substantial but more moderate metabolic response compared to Sakha 104, with numerous significantly upregulated metabolites, including organic acids, sugars, and specialized compounds (Fig. 6D). Malic acid derivative showed significant upregulation, as did a ribose isomer. Quinic acid, a cyclohexanecarboxylic acid involved in the shikimate pathway and phenolic compound biosynthesis, exhibited pronounced accumulation. Shikimic acid showed strong upregulation, consistent with its accumulation in Giza 177 roots and indicating common activation of aromatic compound biosynthesis across some cultivars. Sucrose exhibited significant accumulation, similar to Giza 178 roots, confirming its importance as an osmoprotectant and transport sugar in root drought responses. Downregulated metabolites in Sakha 108 roots included Benzoin, an aromatic compound, suggesting selective regulation within secondary metabolism. D-xylose showed significant downregulation, as did another ribose isomer, indicating selective regulation of pentose sugars with some isomers accumulating while others decline. Glucose exhibited downregulation, similar to Giza 177, suggesting common metabolic responses. DL-Proline showed downregulation in roots, contrasting with its typical upregulation in leaves and suggesting tissue-specific regulation of this important osmoprotectant.

## Discussion

### Differential physiological responses to drought stress among rice cultivars

The physiological responses to drought stress varied significantly among the four rice cultivars examined in this study, reflecting their differential tolerance capacities [45, 46]. Fresh weight, relative water content (RWC), proline accumulation, and plant height measurements revealed distinct stress response patterns, with Sakha108 demonstrating superior drought tolerance characteristics compared to the more sensitive Sakha104 [47]. The observed reduction in plant height and fresh weight under 15% PEG treatment is consistent with previous reports of drought-induced growth inhibition in rice [48]. RWC maintenance is a critical indicator of cellular hydration status and drought tolerance capacity [49]. Our results demonstrated that drought-tolerant cultivars maintained higher RWC values under stress conditions, suggesting more efficient osmotic adjustment mechanisms. The significant accumulation of proline observed across all cultivars under drought stress confirms its role as a key osmoprotectant [50]. However, the magnitude of proline accumulation varied among cultivars, with higher accumulation generally correlating with improved stress tolerance, as previously reported in drought-stressed rice. The correlation matrix analysis revealed strong relationships between physiological parameters, indicating coordinated stress response mechanisms [51]. The PCA of physiological data showed clear separation between control and drought-treated samples, validating the effectiveness of the stress treatment protocol and confirming that 15% PEG successfully induced drought stress conditions suitable for metabolomic analysis.

### Tissue-Specific metabolic responses reveal Root-Centric stress adaptation

The contrasting metabolic profiles between leaf and root tissues revealed fundamental differences in tissue-specific stress adaptation strategies. Root tissues exhibited substantially higher metabolic plasticity (70.4% variance explained in PCA) compared to leaves (53.9% variance), consistent with their role as primary sensors of soil moisture deficit [52, 53]. Roots are in direct contact with soil and serve as the initial perception sites for drought stress, triggering specific signaling pathways that adjust developmental programs to enhance survival [54, 55]. The approximately 20-fold greater metabolic diversity observed in root tissues compared to leaves supports this functional specialization. Previous studies have similarly demonstrated that root and shoot tissues employ distinct metabolic strategies during abiotic stress adaptation, with roots showing greater metabolic flexibility [54, 55]. The hierarchical cluster analysis revealed that leaf metabolomic profiles clustered primarily by treatment status rather than cultivar identity, indicating conserved stress response mechanisms in foliar tissues. In contrast, root tissues displayed more complex cultivar-specific clustering patterns, suggesting that cultivar-dependent drought adaptation strategies are predominantly implemented at the root level. This tissue-specific differentiation has important implications for breeding programs: selection for drought tolerance should prioritize root-based metabolic markers rather than foliar indicators. The Venn diagram analysis identified only three metabolites with universal expression patterns across all cultivars in roots, compared to four in leaves, further supporting the hypothesis of greater metabolic diversity in root drought responses. This finding aligns with observations that root metabolic plasticity underlies functional diversity in stress tolerance across plant species [56, 57].

### Cultivar-Specific metabolic signatures reveal distinct drought tolerance mechanisms

The VIP analysis identified 40 metabolites with scores exceeding 1.0, establishing them as significant contributors to drought stress responses [58, 59]. Among these, 13 metabolites surpassed the optimal discriminatory threshold of VIP > 1.6, representing the primary biochemical factors underlying cultivar-specific stress adaptations. Each cultivar exhibited unique metabolic signatures that correspond to its differential drought tolerance capacities. In Giza177, the elevated accumulation of leucine and DL-serine under drought conditions indicate enhanced branched-chain amino acid metabolism and activation of the tricarboxylic acid (TCA) cycle. Leucine serves not only as a protein building block but also as a signaling molecule regulating stress responses and energy metabolism. The concurrent increase in tyrosine and alanine in this variety suggests broad activation of amino acid biosynthetic pathways, which may contribute to osmotic adjustment and provide carbon skeletons for stress-related metabolite synthesis [21, 25]. Previous studies have reported increased total free amino acids in drought-stressed alfalfa plants [31], supporting the generality of this response across species. Sakha104’s preferential accumulation of Adenosine-5-monophosphate (AMP) and DL-Aspartic acid under drought stress indicates shifts in energy metabolism and nitrogen assimilation pathways. AMP accumulation may signal cellular energy stress, triggering metabolic adjustments to maintain ATP homeostasis under water deficit conditions. Aspartic acid serves as a precursor for several amino acids and participates in nitrogen metabolism, suggesting that Sakha104 responds to drought by modulating nitrogen allocation. Sakha108 demonstrated a distinct metabolic profile characterized by elevated D-gluconolactone and glycerol 3-phosphate levels in leaf tissues, indicating activation of the pentose phosphate pathway and glycerol biosynthesis. These metabolites play critical roles in maintaining cellular redox balance and membrane integrity under osmotic stress. Root-specific metabolic responses revealed additional cultivar differentiation. Giza177 exhibited significantly higher 3-methyladenine concentrations under drought conditions, a compound involved in nucleotide metabolism and potentially in stress signaling pathways. Giza178 showed pronounced accumulation of Cys-Gly, shikimic acid, ribose isomers, galactaric acid, and L-ornithine under drought stress. The elevation of shikimic acid, a key intermediate in the shikimate pathway, suggests enhanced biosynthesis of aromatic amino acids and phenylpropanoid-derived compounds, which are crucial for stress defense [60]. This observation is consistent with previous reports demonstrating increased shikimate pathway activity under drought conditions [61, 62]. L-ornithine accumulation indicates modulation of polyamine biosynthesis, as ornithine serves as a precursor for polyamines like putrescine, spermidine, and spermine, which function as osmoprotectants and membrane stabilizers during drought stress.

### Amino acid metabolism and TCA cycle modulation as central drought responses

The widespread accumulation of amino acids and TCA cycle intermediates across multiple cultivars highlights these metabolic pathways as central to drought adaptation in rice [22, 29]. The volcano plot analyses revealed differential regulation of amino acid profiles, with leucine, alanine, proline, tryptophan, lysine, and phenylalanine showing cultivar-specific accumulation patterns. Amino acids serve multiple functions during drought stress: as osmolytes for osmotic adjustment, as precursors for stress-protective compounds, as nitrogen storage molecules, and as energy sources when photosynthesis is limited [63–65]. The enhanced accumulation of branched-chain amino acids (leucine, valine) observed in several cultivars may reflect increased protein turnover or reduced protein synthesis under stress conditions, providing alternative energy sources through catabolic pathways. The TCA cycle modifications observed in drought-stressed rice reflect metabolic adjustments to maintain energy production under conditions of reduced photosynthetic activity. Citric acid, isocitrate, and succinic acid showed variable accumulation patterns among cultivars, suggesting different strategies for balancing energy metabolism and biosynthetic demands. Previous metabolomic studies on water-stressed plants indicate that although different crops exhibit various drought response strategies, flavonoids, phenolic acids, amino acids, and carbohydrate metabolites are crucial for overall plant drought resistance. This aligns with our findings in rice [66]. The accumulation of TCA intermediates may also reflect their roles as signaling molecules that coordinate stress responses across multiple cellular compartments [67, 68].

### Sugar metabolism and osmoprotectant accumulation drive osmotic adjustment

The widespread proline accumulation observed across all cultivars (1.9- to 3.9-fold increases) reflects activation of the glutamate-derived proline biosynthetic pathway. In plants, proline synthesis proceeds through two enzymatic steps: (1) glutamate is phosphorylated and reduced to glutamate-5-semialdehyde (GSA) by Δ¹-pyrroline-5-carboxylate synthetase (P5CS), the rate-limiting enzyme encoded by *P5CS1* (constitutive) and *P5CS2* (stress-inducible) genes in rice [69, 70]; (2) GSA spontaneously cyclizes to Δ¹-pyrroline-5-carboxylate (P5C), which is then reduced to proline by P5C reductase (P5CR). Under drought stress, proline accumulation results from both increased biosynthesis (via *P5CS2* upregulation) and decreased catabolism (via proline dehydrogenase [ProDH] downregulation) [37, 65]. The cultivar-specific differences in proline accumulation magnitude (Giza 178 > Sakha 104 > Sakha 108 > Giza 177) may reflect genetic variation in P5CS activity, ProDH suppression capacity, or proline transport efficiency. The concurrent accumulation of glutamate precursors and proline in our dataset supports active biosynthetic flux through this pathway. Proline’s multifunctional roles as an osmolyte lowering cellular osmotic potential, as a hydroxyl radical scavenger protecting macromolecules, as a molecular chaperone stabilizing proteins and membranes, and as a cellular redox buffer make it a central mediator of drought tolerance [66]. The strong correlation between proline levels and drought-tolerance indices validates its importance in the cultivars examined.

The pronounced spermine accumulation in Sakha 108 leaves (2.8-fold increase, VIP = 2.2) indicates activation of the polyamine biosynthetic pathway, which proceeds through the following enzymatic sequence: ornithine, derived from arginine via arginase, is decarboxylated by ornithine decarboxylase (ODC) to produce putrescine; putrescine is converted to spermidine by spermidine synthase (SPDS) through addition of an aminopropyl group donated by S-adenosylmethionine (SAM), which is first decarboxylated by SAM decarboxylase (SAMDC); spermidine is further converted to spermine by spermine synthase (SPMS) via addition of a second aminopropyl group [71, 72]. The concurrent accumulation of L-ornithine (identified in Giza 178 roots) supports active flux through this pathway under drought stress. Polyamines function as stress protectants through multiple mechanisms: their polycationic nature enables electrostatic interactions with negatively charged phospholipids, stabilizing membranes under osmotic stress; they scavenge reactive oxygen species (ROS), particularly hydroxyl radicals; they modulate ion channel activity, influencing K⁺/Na⁺ balance; and they regulate gene expression through chromatin modifications and interactions with transcription factors [73]. The cultivar-specific nature of spermine accumulation (prominent in Sakha 108, modest in other cultivars) suggests genetic variation in polyamine biosynthetic capacity or catabolism rates, controlled by genes such as *OsADC* (arginine decarboxylase), *OsODC*, *OsSAMDC*, and *OsSPDS/SPMS* in rice [74]. The 73.1% drought tolerance index of Sakha 108, coupled with high spermine levels, supports polyamine accumulation as a contributing tolerance mechanism.

### Secondary metabolism activation supports stress defense systems

The upregulation of shikimate pathway intermediates and phenylpropanoid-related compounds in drought-stressed rice indicates activation of secondary metabolic pathways that contribute to stress defense [75]. The significant accumulation of shikimic acid in Giza 178 roots and quinic acid in multiple cultivars indicates activation of the shikimate pathway, the primary route for aromatic compound biosynthesis in plants. This pathway converts phosphoenolpyruvate (PEP) and erythrose-4-phosphate (E4P) from primary metabolism into chorismate, the universal precursor for aromatic amino acids (phenylalanine, tyrosine, tryptophan) [76, 77]. Pathway Architecture and Drought Significance: The shikimate pathway proceeds as follows: E4P + PEP → 3-deoxy-D-arabino-heptulosonate-7-phosphate (DAHP) → 3-dehydroquinate (DHQ) → shikimate (via shikimate dehydrogenase) → shikimate-3-phosphate → 5-enolpyruvylshikimate-3-phosphate (EPSP) → chorismate. Shikimic acid accumulation indicates either: increased flux through early pathway steps driven by upregulated DAHP synthase, or temporary buildup at this intermediate caused by downstream metabolic bottlenecks. Phenylpropanoid Connection: Chorismate is then directed into aromatic amino acid biosynthesis, with phenylalanine as the starting point for the phenylpropanoid pathway via phenylalanine ammonia-lyase (PAL). This pathway produces various drought-protective secondary metabolites, including: ( hydroxycinnamic acids (p-coumaric acid, caffeic acid, ferulic acid, sinapic acid)—antioxidants that neutralize ROS and strengthen cell walls through lignification; flavonoids (flavonols, anthocyanins) powerful antioxidants and UV shields; (3) lignin precursors providing structural support and decreasing water loss; and (4) chlorogenic acid and related esters multifunctional antioxidants. Our detection of ferulic acid (upregulated in Sakha 178 leaves) and chlorogenic acid (downregulated in Sakha 104 leaves, implying quick use) confirms active phenylpropanoid metabolism. The cultivar-specific pattern of shikimic acid buildup (Giza 178 > Sakha 108 > others) might reflect genetic differences in: shikimate dehydrogenase activity or regulation, carbon allocation priorities between primary and secondary metabolism, or capacity for phenolic antioxidant production. Mechanistic Integration: The coordinated regulation of shikimate pathway activation with proline, spermine, and organic acid accumulation demonstrates a synchronized deployment of multiple stress-defense mechanisms. Phenylpropanoid-derived antioxidants enhance the ROS-scavenging roles of polyamines and amino acids, creating a multilayered protection system. The tissue-specific pattern of shikimic acid accumulation (mainly in roots) suggests that roots prioritize antioxidant synthesis and structural strengthening, while leaves rely more on compatible solute accumulation for osmotic adjustment. Ferulic acid, chlorogenic acid, and p-coumaric acid, all phenylpropanoid derivatives, showed differential regulation patterns among cultivars. The observed changes in organic acid profiles, including oxalic acid, malic acid, and quinic acid, reflect metabolic adjustments that extend beyond primary carbon metabolism. Oxalic acid, which showed significant upregulation in multiple cultivars, may participate in metal chelation and cellular pH regulation under stress conditions. Previous transcriptomic and metabolomic studies have demonstrated that key metabolic pathways that maintain photosynthesis under drought involve coordinated regulation of primary and secondary metabolites [29]. The integration of amino acid metabolism, TCA cycle modulation, and secondary metabolite production observed in our study supports this holistic view of drought stress adaptation [67].

## Conclusions

This comprehensive GC-MS-based metabolomic investigation revealed profound tissue-specific and cultivar-dependent metabolic adaptations to drought stress in four Egyptian rice cultivars. Root tissues demonstrated approximately 20-fold greater metabolic diversity than leaves, confirming their role as primary stress sensors and sites of genotype-specific adaptation. Variable importance in projection analysis identified 13 high-priority metabolic biomarkers (VIP > 1.6) with strong discriminatory capacity, spanning amino acids, organic acids, sugars, and specialized metabolites. Cultivar-specific signatures included maintenance of energy homeostasis in Sakha 104 (tolerance index: 82.7%), polyamine-based protection in Sakha 108, and amino acid accumulation strategies in Giza 177 and Giza 178. Strong correlations between metabolomic profiles and physiological parameters validated the predictive power of biomarkers for assessing drought tolerance. These findings provide actionable targets for metabolite-assisted selection in breeding programs and reveal that multiple metabolic strategies can confer drought tolerance. Future research should focus on field validation of identified biomarkers, temporal metabolomics across progressive stress intensities, multi-omics integration to elucidate regulatory mechanisms, and metabolic engineering of high-priority pathways. This study establishes a biochemical framework for developing climate-resilient rice cultivars that can maintain productivity under water-limited conditions.

## Supplementary Information

Supplementary Material 1.

Supplementary Material 2.

Supplementary Material 3.

## Data Availability

All data generated or analyzed during this study are included in this published article. The datasets used and analyzed during the current study are available from the S.F.L. on reasonable request.
