# Supplementary material for: Tissue-specific metabolomic profiling reveals cultivar-dependent drought tolerance mechanisms in rice (Oryza sativa L.)
Source: BMC Plant Biol. 2026 Jan 21;26:170. doi: 10.1186/s12870-025-08062-9 (PMC12849649; doi:10.1186/s12870-025-08062-9)
Supplement: Supplementary file 3 — Supplementary Material 3. [file 12870_2025_8062_MOESM3_ESM.docx]

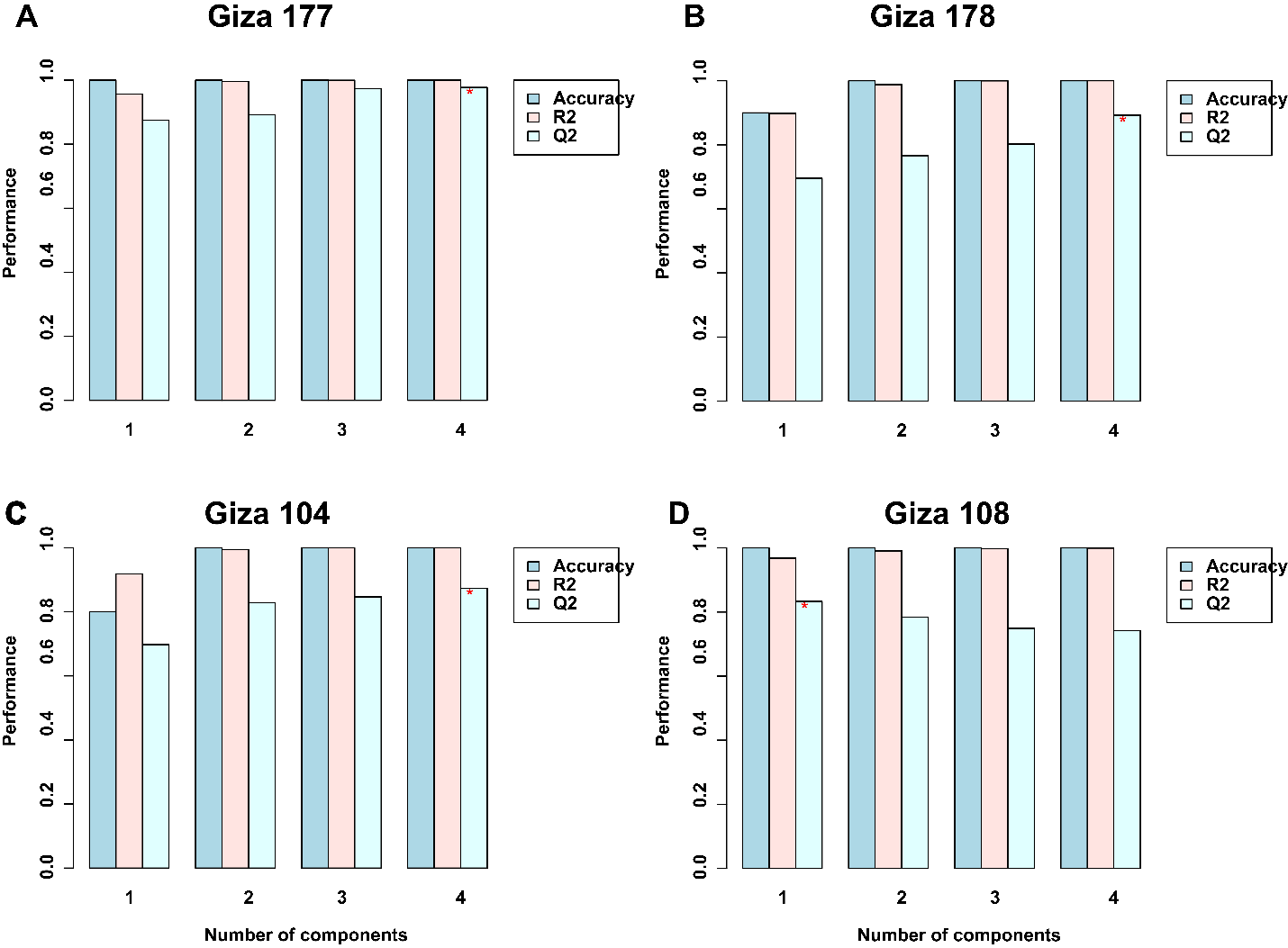


**Figure S1.** Orthogonal partial least squares discriminant analysis (OPLS-DA) model validation for metabolomic data. (**A**) Cross-validation and permutation test results for leaf tissue (A-Giza177, B- Giza178, C-Sakha104, D- Sakha108) showing R² and Q² values exceeding 0.7, confirming model validity and reliability.


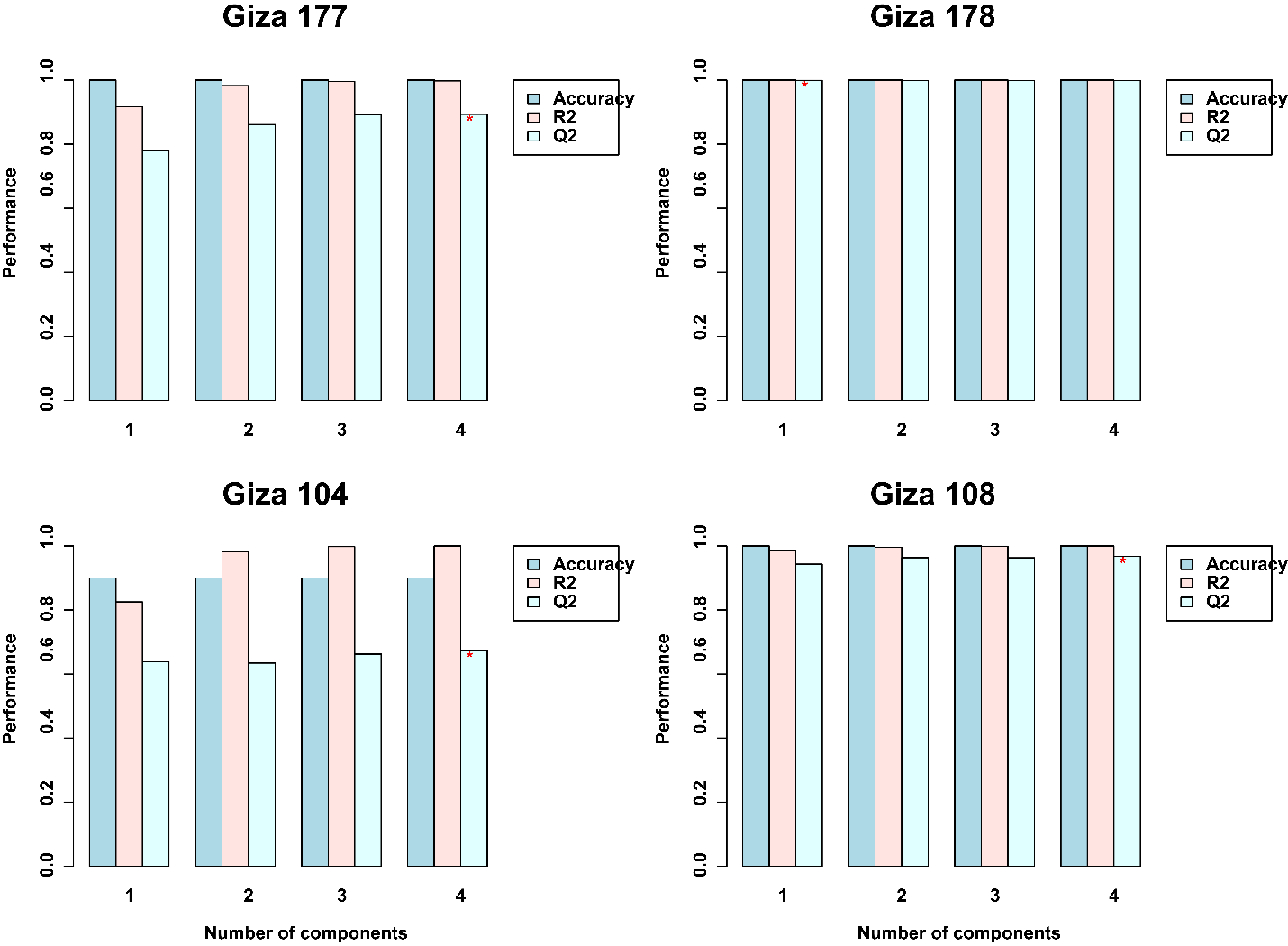


**Figure S2.** Cross-validation and permutation test results for root tissue (A-Giza177, B- Giza178, C-Sakha104, D- Sakha108) showing R² and Q² values exceeding 0.7, confirming model validity and reliability. Quality control samples demonstrated R² values > 0.97, indicating high data quality for metabolomic profiling of four rice cultivars under drought stress conditions.

**Metabolomic Data Quality Assessment and Model Validation**

The reliability of the metabolomic dataset was evaluated using thorough quality control metrics and validation of multivariate models. The performance of the orthogonal partial least squares discriminant analysis (OPLS-DA) model was assessed through cross-validation and permutation testing, focusing on key metrics such as accuracy, R², and Q² values. Quality control analysis indicated high data reliability across all samples. The correlation coefficients (R²) for quality control samples consistently surpassed 0.97, nearing the ideal value of 1.0, which indicates high-quality raw data collection. The strong performance observed in leaf tissue (Figure S1) and root tissue (Figure S2) samples confirms the dependability of the metabolomic profiling method. Multivariate analysis showed clear separation among treatment groups, with samples clustering according to their specific experimental conditions. The four rice cultivars (Giza177, Giza178, Sakha104, and Sakha108) displayed unique metabolomic profiles in response to drought stress, with biological replicates clustering tightly within their respective groups. The clustering patterns observed demonstrate strong experimental reproducibility and validate the effectiveness of drought stress treatments in eliciting cultivar-specific metabolomic responses.
